# Habitat complexity influences selection of thermal environment in a common coral reef fish

**DOI:** 10.1093/conphys/coaa070

**Published:** 2020-09-08

**Authors:** Tiffany J Nay, Jacob L Johansen, Jodie L Rummer, John F Steffensen, Morgan S Pratchett, Andrew S Hoey

**Affiliations:** 1 ARC Centre of Excellence for Coral Reef Studies, James Cook University, James Cook Dr., Townsville, QLD 4811, Australia; 2 Hawaii Institute of Marine Biology, University of Hawaii, 46-007 Lilipuna Rd, Kaneohe, HI 96744, USA; 3 Marine Biological Section, Department of Biology, University of Copenhagen, Strandpromenaden 5, Helsingør, 3000, Denmark

**Keywords:** Behaviour, ocean warming, range shift, teleost fish, temperature preference, temperature threshold

## Abstract

Coral reef species, like most tropical species, are sensitive to increasing environmental temperatures, with many species already living close to their thermal maxima. Ocean warming and the increasing frequency and intensity of marine heatwaves are challenging the persistence of reef-associated species through both direct physiological effects of elevated water temperatures and the degradation and loss of habitat structure following disturbance. Understanding the relative importance of habitat degradation and ocean warming in shaping species distributions is critical in predicting the likely biological effects of global warming. Using an automated shuttle box system, we investigated how habitat complexity influences the selection of thermal environments for a common coral reef damselfish, *Chromis atripectoralis*. In the absence of any habitat (i.e. control), *C. atripectoralis* avoided temperatures below 22.9 ± 0.8°C and above 31.9 ± 0.6°C, with a preferred temperature (*T*_pref_) of 28.1 ± 0.9°C. When complex habitat was available, individual *C. atripectoralis* occupied temperatures down to 4.3°C lower (mean ± SE; threshold: 18.6 ± 0.7°C; *T*_pref_: 18.9 ± 1.0°C) than control fish. Conversely, *C. atripectoralis* in complex habitats occupied similar upper temperatures as control fish (threshold: 31.7 ± 0.4°C; preference: 28.3 ± 0.7°C). Our results show that the availability of complex habitat can influence the selection of thermal environment by a coral reef fish, but only at temperatures below their thermal preference. The limited scope of *C. atripectoralis* to occupy warmer environments, even when associated with complex habitat, suggests that habitat restoration efforts in areas that continue to warm may not be effective in retaining populations of *C. atripectoralis* and similar species. This species may have to move to cooler (e.g. deeper or higher latitude) habitats under predicted future warming. The integration of habitat quality and thermal environment into conservation efforts will be essential to conserve of coral reef fish populations under future ocean warming scenarios.

## Teaser

*Chromis atripectoralis* tolerated lower, but not higher, temperatures when associated with complex habitat structure compared to those under control conditions, which had no access to habitat structure. Understanding how habitat complexity influences thermal selection is critical given the rate of ocean warming and poleward expansions of tropical fishes.

## Introduction

Changing environmental conditions, and most notably increasing temperatures, are having important direct and indirect effects on marine species (Hoegh-Guldberg & Bruno, 2010; Pecl *et al*., 2017; Comte & Olden 2017) and are being compounded by local anthropogenic stressors. The direct effects of increasing temperatures on an organisms physiology are driving shifts in individual behaviour (e.g. Beever *et al.*, 2017), phenology (e.g. Nagelkerken and Munday, 2016) and species distributions (e.g. Feary *et al.*, 2014). These shifts are especially pronounced in tropical marine ecosystems, as tropical species are generally exposed to environmental conditions that are closer to their upper thermal maxima and have fewer thermal refugia than freshwater and terrestrial ecosystems (Comte and Olden, 2017, Pinsky *et al.*, 2019). The direct effects of increasing temperature on physiology are occurring alongside indirect effects, such as the degradation, fragmentation and/or loss of habitat (Robinson *et al.*, 2019). For example, across tropical and temperate reef systems, climate-induced changes in environmental and biological conditions are causing massive reductions in the abundance of key habitat-forming organisms (Hughes *et al.*, 2018b, Ling *et al.*, 2009, Madin *et al.*, 2018, Vergés *et al.*, 2016, Wernberg *et al.*, 2010). Declines in the abundance of formerly dominant habitat-forming organisms (i.e. reef-building corals and kelp forests), and corresponding declines in habitat complexity, can have a profound influence on the biodiversity and functioning of these ecosystems (Graham *et al.*, 2006). Our ability to predict and manage populations under ongoing climate change will require a greater understanding of both the indirect and direct effects in shaping species’ distributions.

Coral reefs are extremely vulnerable to climate change (Walther *et al.*, 2002), due largely to the thermal sensitivities of the dominant habitat-forming species, reef-building corals (e.g. Baird and Marshall, 2002, Hughes *et al.*, 2018a). The increased frequency and intensity of thermal bleaching events over the past few decades (Hughes *et al.*, 2018a) have contributed to widespread and sustained declines in the abundance of corals and a corresponding loss of structural complexity (Bento *et al.*, 2016, Berumen and Pratchett, 2006, Hughes *et al.*, 2018b, Hughes *et al.*, 2017, Loya *et al.*, 2001, McClanahan *et al.*, 2007, Alvarez-Filip *et al.*, 2009). These losses of coral cover and structural complexity are having a dramatic effect on reef-associated organisms (Pratchett *et al.*, 2008, Stella *et al.*, 2011). Those species that rely on live corals for food and/or shelter are the most rapidly and adversely affected by declines in live coral cover (e.g. Pratchett *et al.*, 2008, Stuart-Smith *et al.*, 2018, Wilson *et al.*, 2006), while species that rely on the physical structure of corals typically exhibit protracted declines as the coral skeletons erode and the physical structure is lost (Graham *et al.*, 2006, Pratchett *et al.*, 2011, Wilson *et al.*, 2006).

Marine fishes, like other ectotherms, are particularly sensitive to increasing temperatures, as their rates of physiological and biochemical processes are largely determined by environmental temperature (Fry, 1947), and they generally occupy environments that are already close to their upper thermal limits (Madeira *et al.*, 2012, Rummer *et al.*, 2013, Vinagre *et al.*, 2016). Moreover, tropical marine species tend to have a narrower thermal tolerance range than temperate species as they evolved in relatively constant thermal environments (Tewksbury *et al.*, 2008), and hence exhibit smaller thermal safety margins (Pinsky *et al.*, 2019). Indeed, many low latitude populations of tropical fishes are already living in thermal environments that are near or even above their thermal optima (Gardiner *et al.*, 2010, Nguyen *et al.*, 2011, Rummer *et al.*, 2013), limiting their capacity to cope with local increases in temperatures (Collins *et al.*, 2013, Hughes *et al.*, 2018a, Kerr, 2011). Stuart-Smith *et al.* (2018) reported a restructuring of fish and invertebrate communities following the 2016 coral bleaching event on the Great Barrier Reef due to the direct effects of temperature.

Given the predicted increases in ocean temperatures with ongoing climate change and concurrent habitat degradation, a greater understanding of the preferred and threshold temperatures of coral reef fishes, and the ecological factors that may influence these temperatures, is urgently needed. Further, the relationship between habitat quality and thermal conditions will be imperative in understanding effective restoration and conservation techniques for the retention of future reef fish populations. The objective of this study was to investigate the combined effects of physical structure and thermal environment in shaping habitat choice of a common coral reef fish, *Chromis atripectoralis*. Specifically, this study used an automated shuttlebox system to determine how availability of a complex habitat influenced the preferred and threshold temperatures of *C. atripectoralis*. Given the strong positive associations between coral reef fishes and complex reef structure, we hypothesized that the ecological benefits gained through associating with complex habitat structure would allow fish to select temperatures beyond those preferred under control conditions (i.e. in the absence of complex habitat).

## Materials and methods

### Animal husbandry

The black-axil chromis (*C. atripectoralis*, Pomacentridae) was selected as the model species as they are common across a wide range of latitudes on Indo-Pacific reefs (32°N-32°S, from the Ryukyu Islands, Japan to Northern Australia; Froese and Pauly, 2019). *Chromis atripectoralis* are relatively small bodied (maximum total length, TL: 12 cm) and closely associate with complex coral structures (e.g. branching *Acropora* and *Pocillopora* corals, see Pratchett *et al.*, 2012), making them an ideal species for examining the impact of habitat complexity on thermal preference. *Chromis atripectoralis* were collected from Pioneer Bay, Orpheus Island, Queensland, Australia (18.6161° S, 146.4972° E, annual temperature range: 21–29°C; AIMS 2020) using small barrier nets and hand nets in May and June 2017. Following collection, fish were held at the Orpheus Island Research Station with fresh flow-through seawater for 48 h and then transported in bags filled with seawater with supplemental aeration delivered using a portable air pump and air stone, to the Marine Aquaculture Research Facilities Unit (MARFU) at James Cook University, Townsville, Queensland, Australia. Transport lasted ~3 h, and no mortalities were recorded during this time. Forty-five similar-sized *C. atripectoralis* (mean ± SEM; TL: 5.91 ± 0.16 cm; mass: 11.28 ± 0.74 g) were randomly selected and held in 100 L of aquaria, with a maximum of 10 fish per aquarium. All aquaria were equipped with supplemental aeration and were continuously supplied with filtered seawater maintained at 26 ± 1°C. Fish were fed commercial pellets twice daily and held under a 12:12-h photoperiod. Fish were habituated to laboratory conditions for 2 weeks after which they were each tagged with subcutaneous coloured elastomer (Northwest Marine Technology, Washington, USA) in the dorsal musculature for individual identification and allowed to recover for a minimum of 2 weeks prior to experimentation. The research project was conducted in compliance with the National Health and Medical Research Council (NHMRC) Australian Code of Practice for the Care of Use and Animals for Scientific Purposes, 7th Edition, 2004, and the Qld Animal Care and Protection Act, 2001, and received animal ethics approval from the JCU Animal Ethics Committee Approval Number A2089.

### Preferred and threshold temperatures

To establish the effect of habitat complexity on preference temperature, a modified shuttlebox design was used, in which structurally complex habitat (branching coral skeleton) was added to the centre of one chamber and a structurally simple habitat (coral rubble) of equal volume (~900 cm^3^) was added to the other chamber. In brief, the shuttlebox system is a two-chamber choice system, in which a temperature differential of 1.5°C is maintained between the chambers by water flowing in a clockwise direction in one chamber and counter-clockwise direction in the other chamber (Schurmann *et al.,* 1991). The two chambers (diameter = 35 cm, water depth = 20 cm, volume = 19.2 L) are connected by a 5 cm wide opening allowing the fish to freely move between chambers. A camera (SONY® HDR-XR100E) linked to a custom programme (Laboratories Technology Corp., Andover MA) controlled the rate of temperature change in each chamber by activating or deactivating the appropriate pumps based on the position of the fish. A PC video frame grabber (USB 2.0 DVD maker®) transmitted a video signal to a positioning software (LoliTrack, Loligo Systems®, Tjele, Denmark) which continuously tracked the position of the fish. If the fish was in the warmer of the two chambers, the temperature of the entire system would increase at 6°C h^−1^. If the fish was in the cooler chamber, the temperature of the entire system would decrease at 6°C h^−1^. By moving between the chambers, the fish could actively control the temperature of their environment. An intact skeleton of the branching coral *Acropora nasuta* (~15-cm diameter, ~15-cm height) was used as the ‘complex’ habitat. Rubble (~15-cm diameter, ~5-cm height) was created by breaking up an *A. nasuta* skeleton of similar size. These pieces of coral rubble were then placed on a flat terracotta plate and used as the ‘simple’ habitat. The skeleton branching coral was used instead of live coral, as we aimed to establish the effects of structural complexity, independent of the health and condition of the coral habitat. The *A. nasuta* skeleton allowed fish to occupy space under, above and between coral branches, while the rubble structure was of similar volume but provided fish with limited refugia. To avoid potential problems of tracking fish within the complex structure, we placed a ‘mask’ over the habitat structures. If a fish entered the mask, the LabTech software would use the previous position of the fish until the fish moved outside of the mask. The preferred and threshold temperatures of *C. atripectoralis* were determined: (i) in the absence of any habitat in either chamber, i.e. ‘control’, (ii) with the complex structure in the ‘warmer’ chamber and rubble structure in the ‘cooler’ chamber and (iii) with the complex structure in the ‘cooler’ chamber and rubble structure was placed into the ‘warmer’ chamber. Fifteen *C. Atripectoralis* were used for each treatment, with a different individual being used for each trial (total *n* = 45).

Individual fish were haphazardly selected and allocated to one of the three treatments. The order of treatment (i.e. control or habitat) for habitat trials and the position of structures (i.e. complex and simple structure) were randomized amongst trials. All fish were fasted for 24 h prior to trials to remove the influence of digestive processes on the measurements (Niimi and Beamish, 1974). For each trial, the fish and habitat were placed into the system at 1430 h, and given 1.5 h to familiarize with the system prior to the heating and cooling pumps being activated. Fish were given an additional 17-h learning period prior to data collection. Data collection began at 0900 h the next morning and continued for 5 h (i.e. until 1400 h). Following each trial, the shuttlebox was drained, cleaned and refilled with fresh seawater in preparation for the next trial.

For each treatment, preferred temperature (*T*_pref_) was defined as the temperature where the fish spent the most time (i.e. modal temperature) within each trial. The lower and upper threshold temperatures were defined as the lowest and highest absolute temperatures, respectively; each individual fish experienced when associated with either a complex or rubble habitat. For the control, the lower and upper threshold temperatures were defined as the lowest and highest temperatures experienced by each fish during a trial. For the habitat trials, the proportion of time spent associated with each habitat type (i.e. complex or simple) was calculated for each individual.

**Figure 1:**
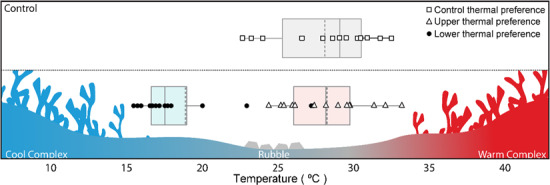
Boxplots representing mean *T*_pref_ (dashed lines), median *T*_pref_ (solid lines), interquartiles, and upper (open triangles) and lower (solid circles) thermal preferences for each individual, for fish occupying the control vs. complex habitat environment

**Figure 2:**
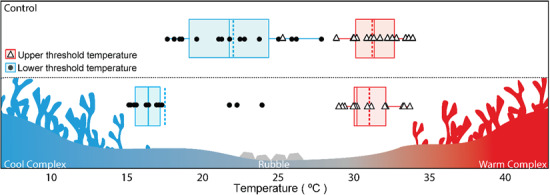
Boxplots representing mean threshold temperature (dashed lines), median threshold temperature (solid lines), interquartiles, and upper (open triangles) and lower (solid circles) threshold temperatures for each individual, for fish occupying the control vs. complex habitat environment

### Data analyses

All analyses were performed in R (Version 3.5.12018, R Core Development Team) using ‘*lme4*’. Generalized linear mixed-effects models (GLMM) using the gamma distribution and ‘log’ link function were used to compare *T*_pref_ and upper and lower threshold temperatures between control and complex or rubble habitats. The most appropriate statistical family and error distribution for each analysis was determined by examining the distribution of the response variable and visually inspecting the residuals for the saturated models. Treatment was used as a fixed effect, and holding tank was included as a random effect. All assumptions were checked by visual inspection of residuals, Shapiro–Wilk normality tests, variance inflation factors and Q-Q plots. Tukey *post hoc* tests were used for all *a priori* analyses. All values are reported as mean ± SEM.

## Results

Lower preferred temperatures (blue) were established when complex habitat structure was placed into the ‘cooler’ chamber, while upper preferred temperatures (red) were established from trials where complex habitat structure was placed in the ‘warmer’ chamber (Fig. 1). The lower (blue) and upper (red) threshold temperatures are the minimum and maximum temperatures experienced by a focal *C. atripectoralis* for each of the treatments (Fig. 2).

**Figure 3:**
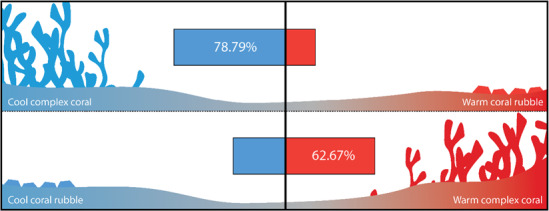
The proportion of time *Chromis atripectoralis* spent associated with structurally complex (i.e. *A. nasuta* skeleton) versus structurally simple (i.e. rubble) habitat within the shuttlebox. The upper panel displays the proportion of time when the complex habitat was positioned in the ‘cooler’ chamber, and the lower panel displays the proportion of time when the complex habitat was positioned in the ‘warmer’ chamber of the shuttlebox

In the absence of any habitat (i.e. control), *C. atripectoralis* avoided temperatures below 22.9 ± 0.8°C and temperatures above 31.9 ± 0.6°C, while preferred a temperature of 28.1 ± 0.9°C (Figs 1 and 2). When the alternative habitat types (rubble versus complex habitat of *A. nasuta*) were added to the shuttlebox, fish preferentially associated with the complex habitat spending 62.7 and 78.8% of each trial associating with the complex habitat, as opposed to rubble (Fig. 3).

When associated with the complex habitat, *C. atripectoralis* would tolerate lower (18.6 ± 0.7°C, *z* = 4.37; *P* < 0.001), but not higher (31.7 ± 0.4°C, *z* = 0.27; *P* = 0.79; Fig. 2) threshold temperatures than control fish. Further, *C. atripectoralis* preferred temperatures of 18.9 ± 1.0°C (*z* = 8.27; *P* < 0.001) or 28.3 ± 0.7°C (*z* = −0.752; *P* = 0.73) depending on the placement of the complex habitat in the ‘cooler’ or ‘warmer’ chamber, respectively (Fig. 1).

## Discussion

Increasing ocean temperatures will have both direct and indirect effects on coral reef fishes (Stuart-Smith *et al.*, 2015), whereby changes in the distribution of fishes relative to thermal environments may be moderated by temperature-induced changes in habitat structure. Here, we demonstrate that at lower temperatures, a common coral reef fish, *C. atripectoralis*, appears to trade-off between the ecological benefits of associating with a complex habitat and physiological costs of occupying a suboptimal thermal environment. In the absence of any habitat, *C. atripectoralis* avoided temperatures <23 and > 32°C, with a preferred temperature of 28.1°C. When associated with the complex habitat, individual *C. atripectoralis* experienced temperatures 4.5°C lower than control fish (i.e. in the absence of any habitat), resulting in a 9.2°C decrease in their preferred temperature. In contrast, we found no evidence that *C. atripectoralis* would experience temperatures above 31.9°C when associated with complex habitat, likely due to the close proximity to their upper thermal limits (i.e. critical thermal maximum CT_Max_). Our results support previous studies that have shown several tropical damselfishes (including *C. atripectoralis*), and cardinal fishes occupy thermal environments that are close to their upper thermal limits (Gardiner *et al.*, 2010, Rummer *et al.*, 2013). While numerous studies have investigated the effects of habitat degradation and loss of structural complexity (e.g. Richardson *et al.*, 2018, Roberts and Ormond, 1987) or changing temperatures (e.g. Donelson *et al.*, 2010, Habary *et al.*, 2017) on reef fishes, few, if any, have considered how habitat availability may affect temperature choice and vice versa (see Matis *et al.*, 2018 for exception). Understanding the nature and magnitude of the costs and benefits of associating with different habitat/s and thermal environments is crucial to predict how populations and distributions of coral reef fishes will respond to future conditions under ongoing ocean warming.

Reductions in live coral and the consequent loss of structural complexity are known to reduce the abundance and diversity of coral reef fish assemblages, with those species that rely directly on corals for food and/or shelter being the most vulnerable (Caley and John, 1996, Coker *et al.*, 2009, Pratchett *et al.*, 2008). While *C. atripectoralis* is considered a facultative coral dweller, a meta-analysis has shown their abundances are relatively insensitive to the loss of live coral (Pratchett *et al.*, 2016). The results of the present study suggest that the preference of *C. atripectoralis* for the complex habitat, although important, may be lesser than the effects of increasing temperature on physiological function and survival. *Chromis atripectoralis* would not tolerate temperatures greater than 31.9°C, even when the preferred complex habitat was available, which is likely due to close proximity of preferred temperatures to the upper thermal limits. However, this response may have been stronger if the complex habitat provided was a live coral colony, given the benefits of live coral versus dead coral skeleton in providing food, moderating competition and predation (Coker *et al.,* 2013). If an obligate coral dweller was examined, there was a threat of predation, or if microthermal refugia were present within the structure. This is supported by a previous study that demonstrated a reduction in maximum oxygen uptake rate and aerobic scope of *Chromis viridis*, the sister species to *C. atripectoralis* with similar preferred temperature (28.9°C), at temperatures above 31°C (Habary *et al.*, 2017). The lack of change in upper threshold temperatures when associating with complex coral structure suggests that behavioural thermal thresholds for this species may be close to upper (acute) critical thermal limits (CT_Max_), as seen in other tropical taxa (e.g. CT_Max_ of ~37°C for *C. viridis*; Habary *et al.*, 2017).

Coral reef fish associations with complex coral structure are well established (Caley and John, 1996, Coker *et al.*, 2009, Pratchett *et al.*, 2008); however, such strong habitat associations could result in an ‘ecological trap’ (i.e. a situation where a given trait is no longer beneficial given the changing environment; Wong and Candolin, 2015). For instance, a focal fish remaining with complex coral structure would benefit from the structural habitat, but risk exposure to suboptimal thermal conditions. Such fish would benefit from a more plastic behavioural response to the changing environment. Ecological traps such as these could cause further exposure to suboptimal thermal conditions, ultimately leading to changes such as lower reproductive output or slower growth, and changes in population structure and dynamics. Indeed, exposure to 31°C for up to 3 months resulted in slower growth for a common coral reef fish, *Acanthochromis polyacanthus* (Munday *et al.*, 2008a), indicating that the growth of some coral reef fish populations may be limited with exposure to suboptimal conditions. Although these effects were not tested here, remaining with complex coral structure and potentially enduring suboptimal conditions could have longer-term effects for coral reef fish populations.

Seawater temperature and habitat structure are widely recognized as two of the major drivers of reef fish communities (Pratchett *et al.*, 2008, Robinson *et al.*, 2019, Stuart-Smith *et al.*, 2009, Waldock *et al.*, 2019), yet are often viewed at different spatial scales. Increasing ocean temperatures have typically been related to shifts in the geographic distribution of reef fishes (e.g. Feary *et al.*, 2014, Sunday *et al.*, 2012), while changes in habitat structure have been related to changes in fish communities within or amongst proximal locations (e.g. Darling *et al.*, 2017, Messmer *et al.*, 2011). The results of this study highlight the need to consider both thermal environments and habitat structure when considering how fishes may be affected by changing environmental conditions. Indeed, the lack of suitable habitat has been suggested to constrain the poleward expansion of some reef fish species (Feary *et al.*, 2014, Munday *et al.*, 2008b). The only other study we are aware of that investigated the effects of temperature on habitat choice of coral reef fishes suggested that exposure to 22, 28 or 31°C influenced habitat selectivity of three species of juvenile damselfishes, and although some differences were reported, the effect sizes were small (Matis *et al.*, 2018).

Global declines in coral cover, and the subsequent reductions in the goods and services that they provide has led to an increased emphasis on coral reef restoration projects to aid in coral reef recovery (Fox *et al.*, 2019, Hein *et al.*, 2017, Rinkevich, 2015). While there are a growing number of approaches to coral restoration (e.g. enhanced larval supply: Cruz and Harrison, 2017, assisted evolution of thermally tolerated corals: van Oppen *et al.*, 2017, growth and outplanting of coral nubbins: Suggett *et al.*, 2019, structural complexity enhancement: Yanovski and Abelson, 2019), all are aimed at increasing the cover of live coral and/or the physical structure of reef habitats. It is often assumed, either implicitly or explicitly, that the provisioning of physical structure will facilitate the recovery of reef fish assemblages (Ladd *et al.*, 2019). However, the physiological tolerances of reef fishes to increasing temperatures are rarely considered. The results of the present study suggest that provisioning habitat structure alone may not be sufficient to restore or maintain fish populations, especially at their lower latitude boundaries, under ongoing climate change scenarios.

Changes in the abundance, diversity and composition of reef fish assemblages have typically been related to changes in coral cover and/or the physical structure of the habitat (Pratchett *et al.*, 2008, Wilson *et al.*, 2006). However, the results of this study suggest that, as oceans continue to warm, the physiological effects of local environmental temperatures are likely to overwhelm any benefit of associating with their preferred habitats and may lead to shifts in the distributions of species to cooler (i.e. deeper and/or higher latitude) habitats. This is particularly important as both theoretical predictions and empirical evidence suggest that many coral reef fish species have limited thermal safety margins as their preferred, and often realized temperatures are close to the thermal maximum (Gardiner *et al.*, 2010, Pinsky *et al.*, 2019, Rummer *et al.*, 2013, Tewksbury *et al.*, 2008). This is critical given the increasing focus on coral restoration efforts to conserve reefs under ongoing climate change (e.g. Boström-Einarsson *et al.*, 2020). There are many obstacles to successful reef restoration (i.e. cost, small spatial scale, high coral mortality; Bellwood *et al.* 2019; Ware *et al.*, 2020), and even if strategies are successful, they may be unable to support associated fish assemblages if the local temperatures exceed the fish species’ preferred thermal temperatures. Further, the trade-off at lower temperatures may influence the poleward range extensions of some fishes. While they may be able to tolerate cooler temperatures, they may not do so in the absence of their preferred (i.e. complex) habitat. An urgent rethinking of conservation actions for coral reefs is required and reinforces the need for action on limiting future increases in global temperatures. Integrating habitat quality with along with thermal conditions will be critical in predicting how fishes will respond to future ocean warming and the potential of restorative techniques for maintaining future fish populations.

## Funding

This work was supported by the ARC Centre of Excellence for Coral Reef Studies at James Cook University (A.S.H.).
